# Editorial: Pathogen transmission at the domestic-wildlife interface: a growing challenge that requires integrated solutions

**DOI:** 10.3389/fvets.2024.1415335

**Published:** 2024-05-21

**Authors:** Saúl Jiménez-Ruiz, Nuno Santos, José A. Barasona, Amanda Elizabeth Fine, Ferran Jori

**Affiliations:** ^1^Departamento de Sanidad Animal, Grupo de Investigación GISAZ, UIC Zoonosis y Enfermedades Emergentes ENZOEM, Universidad de Córdoba, Córdoba, Spain; ^2^CIBIO Research Centre in Biodiversity and Genetic Resources, InBIO Associated Laboratory, University of Porto, Vairão, Portugal; ^3^BIOPOLIS Program in Genomics, Biodiversity and Land Planning, Vairão, Portugal; ^4^VISAVET Health Surveillance Centre, Complutense University of Madrid, Madrid, Spain; ^5^Department of Animal Health, Faculty of Veterinary Medicine, Complutense University of Madrid, Madrid, Spain; ^6^Wildlife Conservation Society, Health Program, New York, NY, United States; ^7^UMR ASTRE (Animal, Santé, Territoires, Risque et Ecosystèmes), CIRAD-INRAE, University of Montpellier, Montpellier, France; ^8^Department of Zoology and Entomology, University of Pretoria, Pretoria, South Africa

**Keywords:** biosecurity, disease management, domestic-wildlife interface, eco-epidemiology, human-wildlife conflict, interaction risks

## 1 Introduction

Wildlife has coexisted with domestic animals in dynamic systems over thousands of years. Domestic-wildlife interfaces are intricate, encompassing physical spaces where wild and domestic species overlap and potentially interact, posing risks of pathogen transmission. The nature of this interface has changed over time and across landscapes, leading to continuous emergence of different conflicts. In addition, human processes that alter ecosystems have led to more interconnected interfaces and increased opportunities for the emergence and spread of shared pathogens (1).

The main goal of this Research Topic was to promote integrative research at domestic-wildlife interfaces globally to characterize and better understand specific eco-epidemiological drivers of pathogen transmission. This knowledge is essential to support subsequent strategies and interventions for disease management and control.

## 2 Organization of the Research Topic and new findings

The fourteen manuscripts comprising this Research Topic of scientific articles cover diverse aspects of domestic-wildlife interfaces. Systematic reviews, original research, case reports, and perspective articles contributed to a deeper knowledge of these interfaces and the eco-epidemiological drivers of pathogen transmission. The majority of contributions focused on domestic-wild mammals (57.1%), and animal tuberculosis (TB), with avian interfaces also explored (28.6%), notably investigating highly pathogenic avian influenza (HPAI) virus H5N1. This breakdown by taxa group and pathogen was similar to literature reviews on these interfaces performed over the last decade (2, 3). A major difference in our Research Topic is the contribution of articles on African swine fever (ASF), reflecting the increased interest in domestic-wild suid interfaces around the world. Disease epidemiology (35.7%) and control (35.7%) were primary areas of investigation, followed by surveillance (14.3%) and predictive modeling (14.3%).

Thompson et al. reviewed the historical perspective of the World Organization for Animal Health (WOAH) on wildlife, and its role in defining the wildlife compartment of this interface and contextualizing the wildlife health framework of WOAH. They articulated a WOAH-led One Health approach with cross-sectoral collaboration to address challenges and assist in preserving wildlife population health and biodiversity conservation. However, communication gaps between the health and environmental sectors, and scarce resources for wildlife health surveillance in many countries, hinders international information sharing and limits availability of epidemiological data on wildlife. This often leads scientists and managers to rely on indirect inference. Hayes et al. performed a scoping review of the scientific literature to illustrate different methodological approaches explored to precisely infer epidemiological outcomes at this complex and dynamic interface. They included a total of 56 research articles published during 2001-2023 with the main focus on mathematical modeling of drivers of disease transmission between domestic and wild hosts. Strengthening wildlife disease surveillance efforts globally requires interdisciplinary collaboration and integration of diverse datasets. By embracing transparency, integrating the One Health approach, and leveraging advanced modeling techniques, the global community may enhance wildlife disease surveillance and mitigate associated risks.

Among domestic-wild mammal interfaces, the relationship among wild boar (*Sus scrofa*) and domestic pigs was highlighted. The interplay between them and disease transmission is a focal point in epidemiological research across different regions. In Corsica (France), Dupon et al. merged different approaches including social sciences, epidemiology, animal husbandry, and geography to estimate the risks of interaction between domestic pigs and wild boar based on pig production practices. They discussed how the information obtained could inform control efforts of shared porcine diseases in extensive farming, not only in Corsica but also at larger territorial scales. In the United States (US), Brown et al. described the state of the knowledge available on ASF, which poses a significant threat to the domestic-wildlife interface and global food security. The authors aimed to prepare the policy context for an integrated and coordinated response against a potential ASF outbreak. Free-ranging or feral suids constituting invasive populations in the US are mostly hybrids of domestic and wild lineages (4), which adds some disease management differences and hinder this response. These animals underscore the need for a holistic approach, considering sociological factors with the same urgency and determination as has been given to the surveillance aspects. In Eastern Poland, the risk factors related to transmission dynamics of ASF virus at the wild boar-domestic pig interface were investigated by Pepin et al. between 2014 and 2019. Results showed that while risk factors related to pig ASF cases did not predict disease detection in wild boar, multiple risk factors for wild boar were able to predict case detection in domestic animals. In addition, they showed that spill over from wild boar to domestic pigs might be more frequent than the reverse, but that the structure of surveillance systems hindered this quantification, highlighting the importance of investigating the movement patterns of both swine species to better understanding transmission routes at this interface. In an experimental study conducted in Spain, Kosowska et al. assessed the potential transmission of an attenuated ASF virus isolate (vaccine candidate) between infectious wild boar and directly exposed naïve domestic pigs, examining the transmission of this viral strain, clinical signs and the level of interaction between *Suidae* species. Authors found that wild boar were successfully protected, did not transmit the virus to susceptible pigs and survived the challenge with the virulent ASF virus isolate during the experiment, without showing ASF-compatible signs or associated viremia. This observation suggests that the presence of wild boar infected with an attenuated virus in ASF-affected areas may reduce the spreading of virulent isolates and virus introduction into the domestic pig husbandry. Altogether, these outcomes may help decision-making related to targeted control actions against ASF in field conditions. These studies collectively underscore the importance of understanding disease dynamics at the domestic-wildlife interface. By combining interdisciplinary methodologies and spatial analyses, researchers aim to enhance our epidemiological knowledge and disease management, ultimately safeguarding and securing animal and human health.

Similarly, TB is another key disease at the domestic-wildlife interface, with humans also included in this complex multi-host system. In Nepal, transmission of *Mycobacterium tuberculosis* complex between elephants and humans was evidenced by Man Rajbhandari et al. They sequenced the whole genome of the strains isolated from two deceased Asian elephants (*Elephas maximus*) and one human. The elephant-derived isolates were closely related to human-derived isolates previously described in the same country, supporting the presence of zooanthroponosis or bidirectional transmission. This highlighted the need for a One Health approach for TB prevention and control at this interface, because it is a serious threat not only to humans and livestock, but also to wildlife species critical to biodiversity conservation. Meanwhile, in Ireland, Chang et al. aimed to better understand local TB transmission between cattle and European badgers (*Meles meles*) through the development of a spatially explicit environmental transmission model that incorporated both within herd/territory and between-species transmission. The model disentangled the relationship between relative badger density and local TB transmission risk and generated the first between-herd R (reproductive ratio) map for TB that identified high-risk areas. This map provided a useful tool for identifying TB hotspots where transmission is driven primarily by badger densities, allowing to direct control strategies. In North-Eastern Lower Michigan (US), on the other hand, Dressel et al. demonstrated the feasibility of TB vaccination of free-ranging white-tailed deer (*Odocoileus virginianus*) via oral baits. The BCG vaccine delivery units included Rhodamine B as a biomarker to subsequently quantify the achievable potential uptake coverage. This strategy demonstrated its scalability as an effective method which could spur further progress toward TB eradication in free-ranging wildlife populations and globally. Overall, these studies highlight the complexity of TB epidemiology, management, and control at the domestic-wildlife interface, as well as the importance of integrating different approaches such as genomics, risk mapping, or innovative vaccination strategies, to mitigate the risk of TB transmission and enhance public health outcomes.

In Cambodia, Porco et al. reported the first case of lumpy skin disease in an endangered banteng (*Bos javanicus*) and the subsequent initiation of a vaccination campaign in domestic cattle to mitigate the challenge of pathogen transmission at the domestic-wildlife interface. In this case, vaccination both supported local livestock-based economies and promoted biodiversity conservation. However, this is only a component of a wider and integrated solution against many other disease threats at the domestic-wildlife interface.

In addition, other studies explored the influence of wild bird communities around domestic avian farms or investigated biosecurity measures and potential risk factors related to the introduction and spread of shared pathogens at this domestic-wildlife interface. Studies on these topics are often more difficult to elaborate and their nature is not commonplace among scientists focusing on both animal and human health, particularly when addressing pathologies resulting from the interaction network that may take place among them. Sánchez-Cano et al. used a camera trapping approach to assess the effectiveness of biosecurity measures in different types of avian farms in Spain. They investigated wild bird communities that visited commercial layer and red-legged partridge farms over a one-year timeframe and assessed the occurrence of interactions. They showed that, independently of the type of farm, the house sparrow (*Passer domesticus*), a potential bridge host for several diseases, was in contact with the surveyed farms as well as with other wild bird species mostly belonging to the order Passeriformes. The most geographically extensive and costly animal health event in the history of the USA occurred in 2022-2023 as a HPAI virus H5N1 outbreak affected more than 70% of both commercial turkey and poultry farms. Knowledge of risk factors for HPAI infection became increasingly relevant because additional domestic flocks, wild birds, and other domestic and wild non-avian species have been infected (5, 6). Patyk et al. and Green et al. conducted two similarly designed case-control studies to identify potential risk factors related to the introduction of HPAI virus H5N1 into commercial meat turkey and table egg operations, respectively. In both cases, data were provided by the United States Department of Agriculture (USDA) Animal and Plant Health Inspection Service (APHIS), with support from the USDA National Agricultural Statistics Service (NASS), as well as by regional/national poultry and turkey organizations. They aimed to compare farm characteristics, management practices and biosecurity methods between case and control farms. Patyk et al. included 66 case and 59 control commercial meat turkey farms from 12 different states. It should be noted that there were a few mistakes in the first published version. Thus, an Erratum (Frontiers Production Office) was also published within this Research Topic with the main goal of amending all detected errors and to update the original research article. Green et al., on their side, used data from 18 case and 22 control commercial table egg farms from eight different States, with the same goals of the previous study. Univariate and multivariable results provided a better understanding of both risk and protective factors for HPAI virus H5N1 infection that can be employed to support science-based updates to prevention and control recommendations to safeguard turkey and commercial table egg farms, respectively, in the United States. Overall, these studies emphasize the critical role of interdisciplinary approaches, robust surveillance systems, and integrated strategies in mitigating disease risks at the domestic-wildlife interface to enhance the prevention of new disease outbreaks and the preservation of both animal and public health, as well as biodiversity.

## 3 Conclusions

In conclusion, this Research Topic of articles provides a very interesting contribution of different studies and perspectives to improve our understanding of pathogen transmission and disease prevention and control opportunities at domestic-wildlife interfaces globally. The diversity of content highlights the multi-faceted nature and the complex dynamics of pathogen transmission between wild and domestic animals and humans. As reflected in the Research Topic, in the global north, increasing wildlife-livestock interfaces are attributed to an expansion of wildlife populations/numbers or points of contact and linked to what are described as direct threats to human productive activities including livestock-based farming or agriculture, among others. Reports from the global south associate human (with their livestock) encroachment on wildlife habitat as inducing the potential conflicts that can facilitate disease emergence among livestock, wild species, and humans. Overall, these findings highlight the importance and complexity of this topic worldwide and the growing need for improving awareness, research, and surveillance, and to develop new interdisciplinary strategies and solutions to address this growing challenge.

## Author contributions

SJ-R: Conceptualization, Formal analysis, Funding acquisition, Methodology, Project administration, Supervision, Validation, Visualization, Writing—original draft, Writing—review & editing. NS: Conceptualization, Validation, Writing—review & editing. JB: Conceptualization, Funding acquisition, Validation, Writing—review & editing. AF: Conceptualization, Validation, Writing—review & editing. FJ: Conceptualization, Supervision, Validation, Writing—review & editing.
